# Autismusspektrumstörungen – Transition vom Jugend- in das Erwachsenenalter

**DOI:** 10.1007/s00115-026-01960-9

**Published:** 2026-04-13

**Authors:** Christine M. Freitag, Ludger Tebartz van Elst

**Affiliations:** 1 Klinik für Psychiatrie, Psychosomatik und Psychotherapie des Kindes- und Jugendalters, Universitätsklinikum, Goethe-Universität Frankfurt am Main, Deutschordenstr. 50, 60528 Frankfurt am Main, Deutschland; 2 Klinik für Psychiatrie und Psychotherapie, Universitätsklinikum Freiburg, Freiburg, Deutschland

**Keywords:** Autismus, Komorbide psychische Störungen, Transition, Gesundheitsversorgung, Sozialrecht, Autism, Co-occurring mental disorders, Transition, Healthcare, Social legislation

## Abstract

Autismusspektrumstörungen gehören mit einer Prävalenz von ca. 0,7 % zu den neuromentalen Entwicklungsstörungen. Sie sind charakterisiert durch Besonderheiten der sozialen Interaktion, der verbalen und nonverbalen Kommunikation, routineartigen und unflexiblen Verhaltensweisen, Sonderinteressen und veränderter Wahrnehmung. Sie manifestieren sich in der ersten Lebensdekade und persistieren in der Regel lebenslang. Damit sind sie wie die meisten anderen neuromentalen Entwicklungsstörungen zwangsläufig nicht nur ein Thema der Kinder- und Jugendpsychiatrie, -psychosomatik und -psychotherapie (KJPPP), sondern auch der Erwachsenenpsychiatrie, -psychosomatik und -psychotherapie (EPPP). Die Transition vom Jugend- in das Erwachsenenalter stellt für viele Betroffenen und ihre Angehörigen, aber auch für Professionelle des Versorgungssystems eine besondere Herausforderung dar. Zentrale Aspekte für die Transition sind einerseits die Behandlung komorbider psychischer, kognitiver und sprachlicher Störungen, zum anderen sozialrechtliche und Versorgungsaspekte. In diesem Beitrag werden diese aus der Perspektive der KJPPP und EPPP beleuchtet.

## Abstract (en)

Autism spectrum disorders (ASD) are neurodevelopmental disorders with a prevalence of approximately 0.7%. People with ASD show characteristic social interaction, verbal and nonverbal communication, routine-like and inflexible behavior, special interests and altered perception. ASD manifest in the first decade of life and usually persist throughout life. Like most other neurodevelopmental disorders, they are therefore inevitably not only a topic of child and adolescent psychiatry, psychosomatics and psychotherapy (CAPPP) but also of adult psychiatry, psychosomatics and psychotherapy (APPP); however, the transition from adolescence into adulthood poses a particular challenge for affected individuals, their caregivers, as well as for professionals in the healthcare and social welfare systems. Key aspects of the transition period include the treatment of comorbid mental, cognitive and language disorders as well as social legislation and healthcare aspects. This article depicts these aspects from the perspective of CAPPP and APPP.

## Hintergrund

Autismusspektrumstörungen (ASS) sind heterogene, chronische neuromentale Entwicklungsstörungen, deren Verlauf sich in Abhängigkeit der eigenen sprachlichen, kognitiven und adaptiven Fertigkeiten, der psychiatrischen Komorbidität sowie unterschiedlicher Umgebungseinflüsse individuell stark unterscheidet. Die zentralen diagnostischen Kriterien sind Besonderheiten in der sozialen Kommunikation sowie das Vorliegen repetitiver, stereotyper Verhaltensweisen, sensorischer Empfindungen sowie intensive bzw. Sonderinteressen mit Beginn in der ersten Lebensdekade. Die Symptome können in Abhängigkeit von Alter, Entwicklungsalter und Geschlecht unterschiedlich ausgeprägt sein. Die aktuellen metaanalytisch gesicherten Prävalenzangaben für Europa liegen bei 0,73 % (95 %-Konfidenzintervall = 0,57–0,91; [29]).

Autismusspektrumstörungen zeigen eine hohe Heritabilität, wobei die genetische Prädisposition in Interaktion mit pränatalen und frühen postnatalen Faktoren wirkt. Neben pleiotropen polygenetischen Faktoren, die auch mit dem genetischen Risiko für Aktivitäts- und Aufmerksamkeitsstörung (Aufmerksamkeitsdefizit‑/Hyperaktivitätsstörung, ADHS) sowie depressive Episoden überlappen [6], sind auch kleine zytogenetische Veränderungen, sogenannte „copy number variations“ [7] und seltene Mutationen [10] als ursächliche genetische Risikofaktoren für ASS nachgewiesen. Bei früh in der Entwicklung erkennbaren Symptomen sowie dem zusätzlichen Vorliegen weiterer neuromentaler Entwicklungsstörungen (NDD), wie z. B. Intelligenzminderung, Sprachstörung oder ADHS, wird von einem höheren genetischen Risiko, z. B. eine Kombination seltener Mutationen und hohem polygenetischen Risiko oder einer stärkeren Penetranz der genetischen Risikofaktoren, ausgegangen als bei einer autistischen Symptomatik, aufgrund derer erst im Jugend- oder jungen Erwachsenalter die Diagnose gestellt wird. Dies zeigt sich auch in entsprechenden Zwillingsstudien [18]. Zudem beschrieb eine registerbasierte, retrospektive Familienstudie aus Schweden bei männlichen Personen mit ASS eine etwas höhere Heritabilität als bei weiblichen [25]. Eine späte Diagnosestellung erfolgt in der Regel bei einem insgesamt geringeren Schweregrad, einer geringeren Komorbidität hinsichtlich zusätzlicher NDD oder auch aufgrund höherer Kompensationsfähigkeiten des betroffenen Individuums oder des sozialen Umfeldes. Die Häufigkeit zusätzlicher komorbider psychischer Störungen sowie Sprach‑, Sprech- und kognitiver Einschränkungen ist bei früher Diagnose in der Regel deutlich höher als bei späterer Diagnosestellung [13, 21]. Die Häufigkeitsmuster der komorbiden psychischen und somatischen Erkrankungen unterscheiden sich ebenfalls zwischen den Altersgruppen [24]. Im Blick auf den Verlauf in das Erwachsenenalter ist aufgrund metaanalytischer Evidenz davon auszugehen, dass nur ca. 20 % derjenigen Personen, die im Kindesalter eine ASS-Diagnose erhalten haben, umfassend selbstständig leben können [20].

Aktuell nehmen die Diagnoseraten bei Jugendlichen und jungen Erwachsenen zu, und es stellen sich vor allem immer mehr weibliche Jugendliche und junge Erwachsene mit der Frage nach einem möglichen Vorliegen einer ASS bei Fachpersonal aus den Bereichen Kinder‑/Jugend‑/Erwachsenenpsychiatrie bzw. -psychotherapie vor [19]. Diese hohe Inanspruchnahme durch jugendliche Mädchen und junge Frauen seit einigen Jahren ist allerdings nicht nur hinsichtlich der Frage nach der Abklärung einer ASS zu beobachten, sondern auch bezüglich Angststörungen und depressiven Episoden, was eine kürzlich publizierte Versorgungsstudie der DAK (Deutsche Angestellten-Krankenkasse) beschrieb (https://www.dak.de/dak/unternehmen/reporte-forschung/kjr-2025-angststoerungen_153340).

Letztlich ist die Ursache dieser erhöhten Inanspruchnahme psychiatrisch-psychotherapeutischer Versorgung durch jugendliche Mädchen und jungen Frauen nicht geklärt. Es sind sowohl pränatal biologische [31] als auch soziale Faktoren denkbar, wie z. B. soziale Ansteckung („social contagion“) durch soziale Medien [14]. In diesem Zusammenhang muss auch darauf hingewiesen werden, dass im Internet verfügbare Informationen zum Thema ASS häufig wissenschaftlich unzutreffend sind [2] und dass die Selbsteinschätzung und das Hilfesuchverhalten von Jugendlichen und jungen Erwachsenen wesentlich durch soziale Umgebungsvariablen beeinflusst wird [1].

Aus internationalen Registerstudien zeigen sich jedoch auch Hinweise, dass neuromentale Entwicklungsstörungen bei Mädchen und jungen Frauen oft spät diagnostiziert werden [28], sodass ein anderer Grund für die erhöhte Inanspruchnahme auch eine bisherige Unterdiagnostik sein kann sein. Weiterhin können Jugendliche und junge Erwachsene mit anderen chronischen psychischen Störungen im Verlauf auch mögliche autistische Symptome, vor allem im Bereich der sozialen Kommunikation zeigen, ohne dass die vollständigen Diagnosekriterien einer ASS gegeben wären [4, 5, 22].

All diese Aspekte sind für die Transition von großer Bedeutung, da einerseits dauerhaft eine psychiatrische und psychosoziale Versorgung vom Kindes- bis in das Erwachsenenalter für Personen mit ASS und ihre Angehörigen notwendig ist, andererseits durch die Abklärung des Verdachts auf das Vorliegen von ASS bei Jugendlichen und jungen Erwachsenen mit anderen psychischen Störungen das Versorgungssystem stark in Anspruch genommen wird und für Erwachsene, die als Kinder mit einer ASS diagnostiziert wurden, nur eingeschränkte Ressourcen zur Verfügung stehen. Im Folgenden werden einige wesentliche Aspekte der psychosozialen Versorgung im Rahmen der Transition vom Jugend- in das junge Erwachsenenalter bei Kindern und Jugendlichen, die eine ASS-Diagnose haben, spezifischer dargestellt.

## Sozialrechtliche Rahmenbedingungen im Vergleich

Die sozialrechtlichen Rahmenbedingungen der Versorgung unterscheiden sich zwischen Kindern und Jugendlichen auf der einen und Erwachsenen mit ASS auf der anderen Seite. Die wesentlichen Unterschiede sind in Tab. 1 aufgeführt.

**Tab. 1:** Fachpersonen und Bereiche der Sozialgesetzbücher

	Kinder und Jugendliche	Erwachsene
*Wesentliche Fachpersonen*	*Ärztinnen/Ärzte sowie Psychotherapeutinnen/Psychotherapeuten im Bereich Kinder- und Jugendpsychiatrie und -psychotherapie*	*Ärztinnen/Ärzte sowie Psychotherapeutinnen/Psychotherapeuten im Bereich Erwachsenenpsychiatrie und -psychotherapie*
Diagnostik	SGB 5 (Krankenkassen)	
Medikamentöse Therapie	SGB 5 (Krankenkassen)	
Psychotherapie	Nur bei komorbiden psychischen Störungen über SGB 5	
*Wesentliche Fachpersonen*	*(Heil‑)Pädagogen, Kinder- und Jugendlichen-Psychotherapeuten, Ergotherapeuten, Logopäden*	*Sozialpädagogen, Fachpersonal in spezifischen Beratungsstellen*
Psychosoziale Intervention	SGB 8 (Kinder- und Jugendhilfe); SGB 9 (Eingliederungshilfe)	SGB 9 (Eingliederungshilfe), SGB 2 und 3 (Bürgergeld, Arbeitsförderung)
Sorgerecht/Betreuung	In der Regel Eltern	Falls Betreuung notwendig: gesetzliche Betreuung/Angehörige

*SGB* Sozialgesetzbuch

Im deutschen Versorgungssystem bestehen hinsichtlich des Übergangs im Rahmen des Sozialgesetzbuchs (SGB) 5 (psychiatrische und psychotherapeutische Versorgung) kaum etablierte Strukturen und auch keine spezifische Finanzierung des entsprechenden Koordinationsaufwandes. Falls ein Kind/Jugendlicher schon Hilfen über SGB 9 erhält, ist hier eine Weiterbetreuung in der Regel möglich, wobei in den Sozialämtern in der Regel auch andere Personen für die unterschiedlichen Altersgruppen zuständig sind. Die Jugendhilfe (SGB 8) entfällt in der Regel ab dem jungen Erwachsenenalter. Sollten Jugendliche beim Übergang in das Erwachsenenalter durch Betreuende unterstützt werden, muss in der Regel durch die Eltern ein entsprechender Antrag gestellt werden.

Grundsätzlich wird die Diagnostik durch die Krankenkassen (SGB 5) übernommen. Im Bereich der Therapie werden die medikamentöse Therapie sowie die teilstationäre und stationäre Behandlung durch die Krankenkassen finanziert. Hinsichtlich der ambulanten Psychotherapie ist es im Kindes- und Jugendalter häufig so, dass diese nur dann durch die Krankenkassen finanziert wird, wenn eine komorbide psychische Störung vorliegt, für die Psychotherapie indiziert ist. Die Finanzierung von Frühintervention im Vorschulalter erfolgt über SGB 9 (Frühförderung), die Finanzierung von Gruppenpsychotherapien erfolgt häufig über SGB 8 (Jugendhilfe) oder SGB 9 (Eingliederungshilfe). Im Erwachsenenbereich werden Psychotherapien (einzeln oder in der Gruppe) in der Regel durch die Krankenkassen finanziert (SGB 5).

Grundsätzlich stehen in allen Bereichen deutlich zu wenig Ressourcen für eine kompetente evidenzbasierte Diagnostik, Therapie und psychosoziale Unterstützung (SGB 9) zur Verfügung. Im Erwachsenenbereich ist die Versorgung regional sogar als prekär zu bezeichnen.

## Prädiktoren für den Verlauf vom Kindes- bis in das Erwachsenenalter

Wie oben beschrieben, besteht eine hohe Variabilität hinsichtlich des Verlaufs von ASS vom Kindes- bis in das Erwachsenenalter. Der wesentliche Prädiktor für den Verlauf sind die kognitiven Fertigkeiten im Kindes- und Jugendalter [20]. Menschen mit ASS und schwerer Intelligenzminderung zeigen die höchste Krankheitslast und den stärksten lebenslangen Unterstützungsbedarf [11, 17].

## Übergang von Schule in das Berufsleben und sozialrechtliche Unterstützungsmöglichkeiten

Eine wesentliche Entwicklungsaufgabe des Jugend- und jungen Erwachsenenalters ist die Verselbständigung, zu der einerseits der Schulabschluss gehört. Zum anderen findet in dieser Zeit der Übergang in eine Ausbildung statt, und es stehen der Auszug aus dem Elternhaus, der Ausbau von tragfähigen außerfamiliären Beziehungen und Partnerschaften sowie das selbständige Alltagsleben an. Zahlreiche junge Erwachsenen mit ASS können einzelne dieser Aspekte aufgrund ihrer kognitiven und sprachlichen Fertigkeiten, der autistischen Kernsymptomatik, zusätzlicher vorhandener komorbider psychischer oder somatischer Erkrankungen nur eingeschränkt oder mit langfristiger Unterstützung erreichen.

Für Kinder und Jugendliche mit ASS bietet die Beschulung in der Regel eine Alltagsstruktur, die von ihnen und ihren Eltern geschätzt wird und die es meist erlaubt, mit den alltäglichen Anforderungen – ggf. mit Unterstützung – zurechtzukommen. Für Jugendliche, die zusätzlich eine mittelgradige oder schwere Intelligenzminderung aufweisen, gibt es im Erwachsenalter die Möglichkeit einer geförderten Beschäftigung. Für Jugendliche/junge Erwachsenen ohne Schulabschluss gibt es zahlreiche Fördermaßnahmen im Rahmen der Berufsvorbereitung oder einer unterstützten Ausbildung. Jugendlichen und jungen Erwachsenen mit Schulabschluss steht grundsätzlich die gesamte Bandbreite an Ausbildungen und Studiengängen zur Verfügung. Auch im Rahmen einer regulären Ausbildung oder eines regulären Studiums gibt es zahlreiche Unterstützungsmöglichkeiten über SGB 2, 3 und 9, die in der folgenden Broschüre zusammengefasst sind: https://www.autismus.de/fileadmin/NEWS/Broschuere_Teilhabe_am_Arbeitsleben_Screenfassung_August_2025__002_.pdf.

## Diagnostische Herausforderungen im Erwachsenenalter

Über das Jugend- in das Erwachsenenalter werden die Alltagsanforderung im Bereich der sozialen Kommunikation und der selbständigen Lebensführung immer komplexer. Bei leichter betroffenen Personen fallen die seit Kindheit bestehenden autismustypischen sozialen Interaktionsprobleme oft erst ab dem Jugendalter so deutlich auf, dass erst dann eine diagnostische Abklärung in die Wege geleitet wird. Typisch ist hier vor allem bei Mädchen und jungen Frauen mit ASS, dass oft zuvor schon eine psychiatrisch-psychotherapeutische Behandlung aufgrund anderer, in der Regel internalisierender Symptomatik, einer Essstörung oder auch aufgrund von nichtsuizidaler Selbstverletzung und Suizidalität erfolgt ist, aber die oft zugrunde liegende ASS aus unterschiedlichen Gründen nicht erkannt wurde [3, 9, 22]. Andererseits stellen sich u. a. auch im Kontext der Diskurse um Neurodiversität vermehrt Personen im Jugend- und Erwachsenenalter erstmalig zur Diagnostik vor. Nicht selten hat dann bereits eine intensive Auseinandersetzung mit der Symptomatik, eine Art „Selbstdiagnose“ mit im Internet verfügbaren Instrumenten und gelegentlich sogar eine Identifikation mit dem Thema „Neurodiversität“ bzw. „ASS“ stattgefunden. Das kann dazu führen, dass die Frage nach einer ASS nicht im diagnostischen Prozess geklärt werden soll, sondern das subjektive Bestreben vor allem auf eine Bestätigung der Diagnose gerichtet ist.

Die klinische Forschung zu diesem Themenfeld der spät diagnostizierten Personen mit ASS steckt noch in den Kinderschuhen. Nach klinischem Eindruck können hier verschiedene Untergruppen identifiziert werden. Zum einen stellen sich erwachsene Personen mit überdurchschnittlichem IQ und oft in Kindheit und Jugend guten psychosozialen Rahmenbedingungen und Kompensationsfähigkeiten vor, deren ASS-Diagnose tatsächlich bis ins Erwachsenenalter nicht erkannt worden ist. Bei einer zweiten Untergruppe ist zwar bei genauer Erhebung der Entwicklungsanamnese die Breite der autismustypischen Symptome in der Vergangenheit erkennbar, der aktuelle Schweregrad im Erwachsenenalter ist aber so gering und das psychosoziale Funktionsniveau so hoch, dass die Diagnosekriterien nicht (mehr) als erfüllt betrachtet werden können. Zum Dritten stellt sich eine Gruppe von Personen vor, die die diagnostischen Kriterien einer Autismusspektrumstörung auch anamnestisch bzw. im Längsschnitt nicht erfüllen. Nicht selten finden sich dann andere neuromentale Entwicklungs- oder Persönlichkeitsstörungen, die das subjektive Gefühl des Andersseins nachvollziehbar begründen können und auch zu interpersonellen Schwierigkeiten führen. Wenn sich junge Erwachsene der dritten Gruppe schon im Vorfeld des diagnostischen Prozesses mit der Diagnose identifiziert haben, kann dies zu erheblichen kommunikativ-interaktionellen Schwierigkeiten in der Beziehung zwischen Professionellen und Patienten führen.

## Fortführung der psychiatrischen und psychotherapeutischen Versorgung im Erwachsenenalter

Hinsichtlich der psychiatrischen, psychotherapeutischen und somatischen Versorgung, die im Erwachsenenalter häufig aufgrund der zusätzlichen psychiatrischen Komorbiditäten notwendig ist [30], sind die folgenden Aspekte zu beachten:

1. Bei Kindern und Jugendlichen werden psychosoziale Interventionen häufig über die Kinder- und Jugendhilfe (SGB 8) oder die Eingliederungshilfe (SGB 9) finanziert. Deshalb besteht hinsichtlich Erwachsenen mit ASS häufig Unsicherheit, ob psychosoziale Therapien über Krankenkassen (SGB 5) finanziert werden. Da im Erwachsenenalter bei den meisten Personen mit ASS auch weitere psychiatrische komorbide Erkrankungen, wie Angst- oder affektive Störungen, vorliegen, ist zumindest in solchen Fällen die Finanzierung über SGB 5 immer möglich.
2. In Deutschland gibt es für Erwachsene keine spezifisch zugelassene pharmakologische Therapie für die Behandlung der autistischen Kern- oder anderer assoziierter Symptome. Die internationale Studienlage zur Wirksamkeit von Psychopharmaka bei Kindern, Jugendlichen und Erwachsenen mit ASS ist in einem systematischen Review mit Metaanalyse zusammengefasst, hier sind auch die entsprechenden Zielsymptome der einzelnen Psychopharmaka aufgeführt [27], die ggf. off-label eingesetzt werden sollten.
3. Diagnosen komorbider psychischer Störungen, die im Kindes- und Jugendalter zusätzlich zu ASS gestellt wurden, zeigen nur eine mäßige Stabilität. Stabil ab dem späten Kindes- bzw. Jugendalter sind in der Regel die individuellen nonverbalen und verbalen kognitiven Fertigkeiten. Die komorbide ADHS-Symptomatik reduziert sich häufig im Verlauf, sodass im Erwachsenenalter als häufigste komorbide Störungen insbesondere Angststörungen, depressive Episoden, selbstverletzendes Verhalten und Suizidalität auftreten [13, 21]. In der AWMF(Arbeitsgemeinschaft der Wissenschaftlichen Medizinischen Fachgesellschaften)-S3-Leitlinie zur Therapie bei Kindern, Jugendlichen und Erwachsenen sind die evidenzbasierten Interventionen bei komorbiden Störungen aufgeführt (www.awmf.org). Sowohl medikamentöse als auch psychosoziale Interventionen sind wirksam, wenn auf die Besonderheiten der sozialen Kognition und Handlungsplanung sowie die individuellen sprachlichen und kognitiven Fertigkeiten der Person mit ASS im Rahmen der Interventionen eingegangen wird.
4. Neben den psychischen Störungen besteht auch ein erhöhtes Risiko neurologisch-internistischer Erkrankungen [24]. Hier ist für das Jugend- und jungen Erwachsenenalter neben metabolischen Erkrankungen insbesondere das Risiko des Neuauftretens einer Epilepsie erhöht [16].

Aufgrund der o. g. Studienlage wird deutlich, dass Erwachsene mit ASS weiterhin einen hohen psychiatrischen und internistisch-neurologischen Betreuungsbedarf haben, der im Rahmen der aktuellen Versorgungslandschaft nicht ausreichend adressiert wird. Zusätzlich bestehen für viele autistische Personen spezifische Barrieren im Hinblick auf die Inanspruchnahme von allgemeinen medizinischen Leistungen. So berichteten in einer Erhebung 80 % der Betroffenen von spezifischen Schwierigkeiten beim allgemeinmedizinischen Hausarztbesuch im Vergleich zu nur 37 % nichtautistischer Personen. ASS-Betroffene überlegen häufig, ob ihre Symptomatik überhaupt einen Hausarztbesuch rechtfertigt (72 %), zudem stellt die telefonische Terminvereinbarung eine hoher Hürde dar [8].

Zusätzlich von entscheidender Bedeutung ist die Integration in die Berufs- und Arbeitswelt, die vielen autistischen Personen nicht unbedingt aufgrund ihrer fachlichen Befähigungen, sondern aufgrund der den Tätigkeiten inhärenten sozialkommunikativen Anforderungen schwerfällt [23]. Ähnlich wie die durch die Schule gegebene Alltagsstruktur werden Arbeit und Beruf bei entsprechender Struktur und Übersichtlichkeit durch Erwachsene mit ASS geschätzt, und ihre alltagspraktischen Fertigkeiten werden hierdurch weiter gefördert. Die beruflich bedingte Alltagsstruktur beugt auch der Entstehung zusätzlicher depressiver oder Angstsymptome vor und fördert die Lebenszufriedenheit [26]. Umso wichtiger erscheint deshalb die koordinierte Unterstützung des Übergangs von Schule in Ausbildung und Beruf sowie danach oder parallel die rasche Integration in die Arbeitswelt. Hier müssen die klassischen Methoden der sozialpsychiatrischen Unterstützung und des Wiedereingliederungsmanagements nach den Möglichkeiten von SGB 2, 3 und 9 zur Anwendung gebracht werden.

Angesichts des Mangels an Fachärzten für Psychiatrie und Psychotherapie besteht hier auch eine zentrale Aufgabe für Psychotherapeuten, die nicht nur in der spezifischen psychotherapeutischen Behandlung, sondern auch im Fallmanagement liegt. Für beide Aufgabenbereiche ist eine umfassende Kenntnis des klinischen Bildes einschließlich psychischer und somatischer komorbider Erkrankungen und der relevanten Differenzialdiagnosen bei Erwachsenen mit ASS mit und ohne Intelligenzminderung wichtig. Denn nur auf dieser Grundlage können die Besonderheiten in Verhalten, Empfinden und Selbstkonzept [12] sowie charakteristische Zustände, wie etwa spezifisch ausgeprägte Stressreaktionen (dissoziative Anspannungszustände, mutistisch-stuporöse Reaktionen, auto- oder fremdaggressive Reaktionsmuster), zuverlässig differenziert und (sozial-)therapeutisch angegangen werden. Neben einer intensiveren klinischen Forschungstätigkeit sind deshalb sowohl die Weiterbildung als auch die Erarbeitung spezifischer (sozial-)therapeutischer Konzepte gerade für Personen im Übergang zwischen Jugend- und Erwachsenenalter von hoher Bedeutung [15].

## Fazit für die Praxis

- Die Weiterführung der psychiatrisch-psychotherapeutischen und somatischen Behandlung sowie die Koordinationsarbeit hinsichtlich sozialpsychiatrischer und beruflicher Unterstützung bei Autismusspektrumstörungen (ASS) sind aktuell ungelöste Aufgaben des Versorgungs- und Teilhabesystems.
- Wünschenswert wäre eine Bündelung und Integration empirisch fundierter psychiatrisch-psychotherapeutischer Behandlungsmöglichkeiten mit den entsprechenden psychosozialen Unterstützungsmöglichkeiten über die Transition vom Jugend- in das Erwachsenenalter.
- Hinsichtlich der geplanten Überarbeitung der Sozialgesetzbücher (SGB) können folgende Maßnahmen empfohlen werden: Eine Klare Zuordnung der Finanzierung der psychosozialen Interventionen bei ASS im Kindes‑, Jugend- und Erwachsenenalter zu nur einem einzigen SGB unabhängig von den kognitiven Fertigkeiten („Intelligenzminderung“/„geistige Behinderung“) der Betroffenen sowie die Einführung und Finanzierung eines Fallmanagements.
